# Extracts and Gleanings

**Published:** 1871-12

**Authors:** 


### OAKUM AND CARBOLIC ACID AS AN ANTISEPTIC DRESSING.

Mr. Lister states, that having read reports from various quarters of the efficacy of oakum, he has lately put it to the test with granulatory sores, where, if it should happen to fail, no mischief would result; and he has found it to more than answer his expectations. The reason for its superiority over oily cloths is, he thinks, readily intelligible. Each fibre of the oakum is imbued with an insoluble vehicle of the antiseptic, so that the discharge in passing among the fibres cannot wash out the agent, any more than it can when flowing beneath the lac plaster to a narrow strip of which an individual oakum fibre is fairly comparable. In some points of view, oakum was even superior to the lac plaster. When the latter is left for several days together, the dis-

45

charge, even though small in amount, soaking into the absorbing cloths, loses the carbolic acid it had received from the plaster, and putrifying from day to day assumes an acrid character, and sometimes produces troublesome irritation of the skin. This is of course avoided by the oakum. Again, the lac plaster being quite impermeable to watery fluid, keeps the skin beneath moist, and in fact covered with a weak watery solution of carbolic acid, which perhaps insinuates itself more or less beneath the "protecting," and maintains a light stimulating influence upon the parts beneath it. But oakum, draining away the discharge as fast as it is formed, avoids this source of disturbance. The result is, that if a granulatory sore be thoroughly washed with an antiseptic lotion, and covered with "protecting" and a well-overlapping mass of oakum secured with a bandage, a dressing is provided which nearly approaches the idea Mr. Lister has long had in view. Mr. Lister's "protecting" above mentioned is made by varnishing oiled silk on both surfaces with copal varnish, which renders it considerably less permeable to carbolic acid, and then it is brushed over with a mixture of starch and dextrine to give it a fibre of material soluble in water, so that it becomes uniformly moistened when dipped into the antiseptic solution. It may be obtained of the Apothecaries' Society, Virginia Street, Glasgow. When it is not at hand, common oiled silk may be used as a substitute for it, if smeared with an oily solution of carbolic acid and used in two layers to make up for its inferior efficacy.—*British Medical Journal.*

### PART II.

### Abridged Extracts and Gleanings from our Exchanges

*MULTUM IN PARVO.*

Diagnosis of Phantom Abdominal Tumors.—In an interesting clinical lecture published in the Medical Times, Dr. J. M. De Costar states that the most efficient mode of diagnosticating phantom tumors of the abdomen is by the use of an anæsthetic. When under the influence of ether, a phantom tumor must disappear, and thus remove all doubt as to the nature of the case. He says, "not in a single instance have I found these apparent tumors remain when the patient is under the influence of ether or chloroform, though they re-appear, and quickly, when he passes from under its influence." Thus, we can control the diagnosis not only of phantom tumors, but muscular contractions of the abdomen, not unfrequently feigned, and which high authority declares a source of difficulty almost impossible to get over."—*Detroit Review of Med.*

An Effective Injection in Gleet.—A writer in the London Medical Press and Circular recommends, as an effectual remedy in gleet, an injection composed of tincture opii, dr. j.; tincture catechu, dr. jss.; mist. acaciæ, oz. ij.; to be used twice daily.

To Disguise Ol. Terebinthini.—I have found the taste of turpentine better disguised in the following prescription than in any way I have used it:

R.—Ol. Terebinth, . . . . dr. ij.
Camp. Spt. Lavender, . . . dr. iij.
Syrup. Simp., . . . . . oz. i.
Mucilage, G. A., . . . . . oz. iij.—M.
S.—Tablespoonful every four or six hours.

C. B. Galloway, M. D.

On the Treatment of Cancer of the Neck of Uterus by Bromine.—Dr. Wynn William, in a paper read before the meeting of the British Association, commenced by making some remarks on the spontaneous removal of malignant tumors, from the study of which he was led on to the injection of bromine into cancerous tumors of the uterus and other parts. He stated that the eight cases published in the last volume of the "Obstetrical Transactions" still continued well. He entered into full details as to the manner of injecting these deposits, and the care required in the use of bromine both as an injection and application, and that before its use the surrounding parts should be well protected by a solution of soda. He exhibited the various instruments he had made for the injection of bromine. He gave the history and successful treatment of a well-selected case of medullary carcinoma of the uterus in the state of disintegration and ulceration by this method. He also gave the particulars of a case of epithelioma of the lower lip which had been previously removed by operation. On the return of the disease the patient was sent to Dr. Wynn Williams, who by two injections of bromine caused the entire and so far permanent removal of the disease.—*Lancet.*

GASTRALGIA.—Dr. J. Hale (Am. Practitioner,) recommends the following:

R.—Argenti Nitratis, . . . gr. xv.

Bismuth subnit., . . . . dr. ij.

Ext. Belladonna, . . . gr. v.—M.

Ft. [illegible] No. xv.

S.—One a-half hour before each meal.

CHLORAL IN SUPPOSITORIES IN PUERPERAL CONVULSIONS.—G. F. Whidborme reports, in the Lancet, the following case: "Mrs. Jane C——, aged forty, soon after the birth of her seventh child, suffered with severe and constant uterine pains, which induced convulsions of a most fearful class. After all the usual remedies were made use of without any permanent effect, I had recourse to the chloral hydrate suppositories: Chloral hydrate, one drachm; hard soap, two scruples; honey, sufficient quantity. After using two of the suppositories, she had no more convulsions. She had four hours' good sleep, from which she awoke convalescent."—*Nashville Medical Journal.*

*Remedy for Poisoning from Poison Oak.*—

R.—Acid Carbolic,
Sodæ Sulph., - - - aa. scru. j.
Adipis, - - - - - - oz. j.—M.
S.—Apply two or three times a day.
It may be also used for foul ulcers, slow healing wounds, etc.

Earache in Children.—Dr. Anstie, after detailing (The Practitioner, October. 1871) the particulars of an attack of auricular herpes zoster in his own person, says that the idea has occurred to him that a large proportion of cases of earache, which are generally set down as examples of suppurative inflammation of the meatus auditorius, are in reality nothing but neuralgic herpes, depending on an affection of the auriculo-temporal nerve. In the earache to which children are liable, the pain often comes on in the first instance violently,—the patient cries out with the severity of the suffering; but then there are remissions, amounting often to complete intermission. Morever, it is but rarely that a well-defined discharge of pus is observed, even when relief to the pain has been somewhat suddenly obtained, as is not unfrequently the case. On the contrary, there is frequently found a shedding of small scales, either dry or moistened with a little bloody sanies, from the lining of the meatus, a few days after the cessation of the pain,—in fact, what looks like the dead epidermis of vesicles that have faded. The whole course of events seems, he says, to correspond much more nearly with that of auricular neuralgic herpes than with that of common abscess of lining membrane of the meatus.

Herpes zoster occurring in children on other parts of the body is not attended by much neuralgic pain, and the occurrence of severe pain in the auricular form of the disease is difficult to explain. Dr. Anstie suggests that it may be due to the fact that superior and inferior maxillary nerves are the first of all sensory nerves in the body to exhibit any tendency to pain of a grade approaching to the severity of true neuralgia; and the reason of this is, possibly, the depressing and exhausting influence exerted upon them by the growth of the teeth.—*Philadelphia Medical Times.*

Dysmenorrhœa.—Dr. J. Hale (Am. Practitioner) gives the subjoined:

| | | |
|---|---|---|
| R.—Tr. Camph., | . . . . | dr. v. |
| Tr. Opii, | . . . . . | dr. ij. |
| Tr. Belladona, | . . . . | dr. j.—M |

S.—Tea-spoonful every hour until the pain is relieved.

*Pur t is Vulvæ.*—It is stated (Gazette des Hopitaux, January 8, 1869,) that this annoying affection is often entirely cured and always diminished by a lotion consisting of five parts of corrosive sublimate dissolved in fifty parts of alcohol. A tea-spoonful of this solution is to be diluted with a pint of tepid warm water, and applied as a wash to the parts several times in the day.

OVARITIS.—Dr. J. Matthews Duncan, in the course of an article contributed to the Edinburgh Medical Journal for September, says that, although unable to give a good idea of the frequency of ovaritis, he is convinced that it is a more common disease than is generally supposed. He says that three forms have been described, —1. Parenchymatous; 2. Follicular; 3. Peritoneal,—but that he is unable to see any real distinction between the parenchymatous and the follicular disease, and that the peritoneal form is out of the question, since the ovary has no peritoneal coat. He admits, however, that peraoophoritis, or inflammation of the adjacent peritoneal membrane, may occasionally occur. Ovaritis may be acute or chronic: the former may terminate in resolution, or its end may be complicated by paraoophoric adhesion or abscess, or true ovarian abscess, or it may end in the chronic form of the disease; the latter may last for several years. One ovary may be affected, or both, or the disease may affect the right and the left ovary alternately. The organ may be enlarged to the size of a hen's egg. Ovaritis is only to be made out by physical examination. Palpation of the hypogastric region will reveal a feeling of fulness or tightness over the affected gland. The ovary may often be felt through the vagina, and, when inflamed, it is exquisitely tender, round, and more or less enlarged. When the ovary is bound down by adhesion, it may sometimes be difficult to distinguish it from the fundus of a retroflected uterus; but the distinction is generally possible if a uterine sound be used. Ovaritis is more frequently accompanied by too profuse menstruation than by amenorrhœa, is occasionally attended with leucorrhœa, and is not inconsistent with fertility. It is extremely frequent in the newly-marred, and in others it may be produced by excessive sexual intercourse. Dr. Duncan does not recommend any peculiar or special treatment.

ANASARCA.—Dr. J Hale (Am. Practitioner) uses the following:

| | | |
|---|---|---|
| R.—Tr. Digitalis, | . . . . . | oz. j. |
| Potass. Iodide, | . . . . . | dr. iij. |
| Vin. Colchici Rad., | . . . | dr. ij. |
| Syr. Sarsap. Comp., | . . - | oz. iij. |
| Aqua Puræ, | . . . . . | oz. ij.—M. |

S.—Tea-spoonful 3 or 4 times a day. The bowels to be freely moved every 3 or 4 days with pulv. jalapæ comp.

Dr. H. has used this prescription in several obstinate cases with complete success. It is also, valuable in other forms of dropsy.

THE TONGUE.—Dr. J. M. Scudder, (Electic Medical Journal,) thus writes of it:

"If the tongue is heavily coated at its base with a yellowish-white fur, we know that there are morbid accumulations in the stomach; and we have to determine between the speedy removal by emesis, the slower removal by the Alkaline Sulphites, or the indirect removal by catharsis.

If the tongue is uniformly coated from base to tip with a yellowish fur, rather full, moist, we have the history of atony of the small intestine, and we give Podophyllin, Leptandrin, and this class of remedies with considerable certainty.

If the tongue is elongated and pointed, reddened at tip and edges, papillæ elongated and red, we have the evidence of irritation of the stomach with determination of blood. The therapeutics is plain: get rid of the irritation *first*, and be careful not to renew it by harsh medication.

Again, we have a tongue that might be designated as *slick*. It is variously colored, but it looks as if a fly should light on it he would slip up and break his neck. It is the evidence of a want of functional power, not only in the stomach and bowels, but of all parts supplied by sympathetic nerves. We treat such a case very carefully, avoid all irritants, and use means to restore innervation through the vegetative system of nerves.

The tongue tells us of acidity and alkalinity of the blood, and in language so plain that it cannot be mistaken.

The *pallid* tongue, with white fur, is the index of acidity, and we employ an alkali—usually a salt of soda, with a certainty that the patient will be benefited. Indeed one who has never had his attention directed in this way, would be surprised at the improvement, in grave forms of disease, from one day's administration of simple Bicarbonate of Soda.

The *deep-red* tongue indicates alkalinity, and we prescribe an acid with a positive assurance that it will prove beneficial. Grave cases of typoid fever and other zymotic diseases, presenting this symptom, have been treated with Acids alone, and with a success not obtained by other means. But it makes no difference what the disease is, whether a recent diarrhœa, or a grave typhoid dis-entery, if there is the deep-red tongue, we give Muriatic Acid with the same assurance of success.

Impairment of the blood—sepsis—is indicated by *dirty* coating, and by dark-colored fur—brownish to black. When we have either the one or the other we employ those remedies which antagonize the septic process.

The bitter tonics are indicated by fullness of tissue, with evident relaxation, impairment of cirulation and muscular movement. The same condition will be an indication for iron. We give Tincture of Chloride of Iron if the tongue is red, Iron by Hydrogen if the tongue is pale.

The pale, trembling tongue is a very good indication for the Hypophosphites.

The pale bluish tongue, expressionless, is the indication for the admistration of Copper.

*Puerperal Convulsions.*—Dr. C. B. Hall, (Cincinnati Lancet and Observer) reports a case of puerperal convulsions, in which he gave dr. i. of pure chloroform, and gtts. iv. of croton oil. The bowels were evacuated. Ordered the head to be shaved and ice applied continuously to occiput. Patient had but two slight convulsions after the treatment was instituted. The Doctor calls attention to the use of chloroform by the stomach, as of "magical" influence, in arresting the convulsions.

Chronic Diarrhœa.—Dr. J. Hale (Am. Pratitioner,) employs the following:

R.—Argenti Nitratis, . . . gr. x.
Bismuth Subnit., . . . . dr. j.
Pulv. Opii, . . . . gr. vj.—M.
Ft. Pil, No. xv.
S.—One 3 times a day.
R.—Acid hydrochlor., diluti, - . . oz. i.
Tr. opii, Tr. Nux Vomica, . . aa dr. iv.
Tr. Zingb., . . . . oz. i.—M.

S.—Tea-spoonful, in water, three times a day. This prescription Dr. H. has found very efficient in cases attended by indigestion.

*Beneficial Effects of Injection of Chlorate of Potash in Treatment of Dysentery after Failure of Opinm.*—It is stated in the *Bericht. d. Krankenanstalt, Rudolph Stiftung* 1867, that Lobel treated, for three entire days, a dysenteric patient, 23 years old, with preparations of opium, by the mouth and in the form of enemata, but without any beneficial result. The discharges still continued of a decidedly bloody, dysenteric character. On the fourth day of the disease, he gave injections consisting of chlorate of potash, scruples j. to two ounces of warm water. The discharges, though still thin, lost immediately their bloody aspect, and assumed a feculent appearance. Under a continuance of the same remedy the stools became, finally, entirely natural. Subsequently Dr. L. treated in a similar manner another case of dysentery, and with similarly speedy good results.—*Centralblatt f. d. Med. Wissenchaften, December*, 1868.—*Am. Jour. Med. Sciences.*

*Gaseoas Distension of the Bowels.*—Dr. G. K. Smith, (Medical World.) speaks favorably of the Faradic current for the relief of gaseous distension of the bowels and especially of the colon.

*Sun-Flower Seed in Hooping-Cough.*—Dr. O. C. Farguhar, (Med. and Surg. Reporter) states that during an epidemic of hooping-cough, in 1859, he had exhausted the list of remedies without benefit to his patients, when, at the suggestion of an old German lady, he was induced to use sun-flower seeds. He procured about one pound of the seeds, bruised them thoroughly in an iron mortar, and putting the seeds in a bottle poured upon them three pints of whisky, and left to digest for seven days. To four ounces of this tincture, he added a-half pint of sweetened water. Of this, a dose for a child one year old, was a table-spoonful every three hours—or oftener. This simple remedy, he affirms, had given him more satisfaction, in hooping-cough, than any other.

*Lotion for Chronic Ulcers.*—The following is recommended:

R.—Carbolic Acid, - - - f. oz. ij.
Linseed Oil, - - - - f. oz. iv.

S.—Saturate lint with this, and apply to ulcer. To be renewed twice, or oftener, a day.

In addition to this, in such cases, we give iodoform and iron in pillula form.

Pinus Canadensis.—Several of our physicians have been trying the fluid extract of the pinus canadensis, and report thus far very favorably as to its merits. We have used it quite freely as a local application in the treatment of spongy and ulcerated conditions of the os uteri, and to a limited extent in the treatment of nasal catarrh. Our experience is satisfactory. For vaginal applications it may be used diluted with glycerine; for nasal catarrh, largely diluted with water. Its action is much the same as that of tannin for these purposes, but its properties as an astringent seem modified by the peculiar vegetable—terebinthinate—property of the hemlock.—*Cincinnati Lancet and Observer.*

*Carbolized Gargle for Diphtheria, Tonsillitis, &c.*—Carbolic acid, 20 minims; acetic acid, ½ drachm; honey, 2 fluid ounces; tincture of myrrh, 2 fluid drachms; water, 6 fluid ounces. The carbolic and acetic acids to be well shaken together before the other ingredients are added.—*Charles Sedgwick, Jr.*

*Carbolized Mixture for Zymotic Diseases.*—Carbolic acid, acetic acid, of each, 1 drachm to 1½ fluid drachms; tincture of opium, 1 fluid drachm; chloric ether, 1 fluid drachm; water, 8 fluid ounces. A table-spoonful every four hours, until the fever has subsided.—*Dr. Alex. Keith.*

CHLORFORM AND CHLORAL IN PUERPERAL CONVULSIONS.—Dr. Hobbs, of Carthage, in a paper on "Chloroform and Chloral in Puerperal Convulsions," collates important facts which indicate that the new anæsthetic promises well in all varieties of labor, since it entirely relieves pain without interfering in the least with uterine contractions. From one to three drachms, given in doses of fifteen grains every fifteen minutes, have been found sufficient to induce the desired result. If further experience proves fully its utility and safety, it must eventually supplant chloroform as an obstetric anæsthetic on account of the greater convenience in its use. The employment of this agent in eclampsia has produced most happy results, and the rapidly increasing records of experience in its use in such cases will probably soon rank it as the remedy *per excellence.—Trans. Ind. State Med. Society.*

SPUPERIOR ITCH REMEDY.—The following formula furnishes the very best remedy for itch the writer has ever employed. He is indebted to Dr. W. C. Moore, of Atlanta, for the formula:

| | |
|---|---|
| R.—Simple Cerate, | |
| Sulphur, . . . . | aa. oz. ij. |
| Bicarb. Sodœ, . . . . . | oz. i. |
| Ungt. Hydrarg, . . . . . | dr. ij.—M. |

S.—Smear a small quantity over the diseased parts every night. Wash well with soft soap and water before each application.

*W. T. G.*

CONIUM IN MATITIS.—A German obstetrician (Practitioner) highly recommends extract of conium, in small doses, repeated several times daily, for the resolution of inflammation of the breast, from stasis of the milk. He reports several cases of cure. Care should be used in procuring an active article. We have used the preparations of conium more or less in a variety of diseases for upward of forty years, and we do not recollect to have derived any well marked benefit from them in a single case.

SPIRIT OF SANDAL WOOD.—(*For Gonorrhœa*).

| | |
|---|---|
| Take of Oil of Sandal Wood, . . | oz. i. |
| Alcohol, . . . . | oz. ii. |
| Oil of Cinnamon, . . . | ℳ. xxv.—M. |

Dose—one to two drachms three times a day.—*Dr. Henderson.*

*Mixture of Quinine and Sulpho-Carbolate of Sodium.*—Quinine sulphate, 1 grain; sulphuric acid, 5 minims. Dissolve, and add to the solution of sodium, sulpho-carbolate, 20 grains; in water, 1 fluid ounce.

Hydatid Tumors Treated by Electrolysis —Drs. Fagge and Durham, have treated successfully eight cases of hydatid disease of the liver by electrolysis. The operation was performed as recommended by Dr. Althus. In each case two needles were passed into the tumor and were connected with the negative pole of a modified Daniell's battery of ten cells. The positive pole, terminating in a moistened sponge, was placed upon the surface of the abdomen. The current was allowed to pass through for a period varying from ten to twenty minutes in different cases. The needles were then withdrawn. The operation in most instances was followed by a rapid diminution of the tumors.

Suppositories for Piles —

| | | |
|---|---|---|
| R.—Butyr. theobromæ, | . . . | oz. i. |
| Iodoformi, | . . . . . . | dr. i. |
| Ft. suppositor, | . . . . | viij.—M. |

[*New Remedies.*

Chloride of Ammonium in Hepatitis and Abscess of the Liver.—Dr. Stewart recommends (Brit. and For Med. Review,) this drug almost as a specific in the treatment of hepatitis and abscess of the liver. He also observes that it will be serviceable in all cases of hepatic diseases, whether of organic or functional derangement. If should be given after the subsidence of the acute symptoms, in doses of 20 grains night and morning. Dr. S. used the remedy in thirty-one cases, without a single fatal result.

Hair Wash (*Best*).—

| | | | |
|---|---|---|---|
| R.—Glycerine, | . . . . . | oz. | ij. |
| Tincture of Myrrh, | . . . . | oz. | j. |
| Cologne, | . . . . . | oz. | j. |
| Tincture of Cantharides, | . . | oz. | ss. |
| Distilled Water, | . . . | oz. | xxiv.—M. |

[*Pharmaceutical Journal—New Remedies.*

*Propylamin in Rheumatism of the Uterus.*—A writer in the Indiana Journal of Medicine, gives the case of a lady who, having had rheumatism in various parts of the body, had, upon one of his visits, "severe pain in the region of the uterus." Extreme soreness and tenderness accompanied the pain low down in the iliac region. After trying a number of remedies without success, and believing the pain due to rheumatism, he gave propylamin with relief. Putting the patient upon its use, she improved very rapidly and is in better health than for years before.

TREATMENT OF CHOLERA AND CHOLERA MORBUS.—Dr. F. Xavier de Rolette, of Pittsburg, writes us that small doses of tartar emetic have proved very efficient in his hands in treating the above diseases. He says: "I consider this medicine a preventive of the cholera. The last time that this plague visited the United States, I was in Rochester, New York. I then published some remarks in the Rochester Advertiser on the efficacy of emetics in the treatment of the cholera, and also attended several cases with complete success. My remarks were published in a Kingston paper, and from thence found their way into the London paper. Since then the emetic has been generally used in that city, as I see by an account in Braithwaite, vol. 52, page 274."—*Exchange.*

POOR MAN'S PLASTER.—

| | | |
|---|---|---|
| Take of Beeswax, | . . . . . | oz. i. |
| Tar, | . . . . . | oz. iii. |
| Resin, | . . . . . | oz. iii. |

To be melted together and spread on paper or muslin.—*New York Druggists' Circular—New Remedies.*

*Hooping-Cough.*—Dr. Miller recommends (Am. Practioner,) the Bromide of Ammonium in combination with tincture veratrum ver., as highly efficacious in hooping-cough.

*Formula for Phthsis.*—Dr. C. B. Hall (Canada Lancet,) recommends the following for consumption:

| | | |
|---|---|---|
| R.—Butyrie, | - - - | oz. ij., drs. vj. |
| Vitell Ovi, | - - - - | No. 1. |
| Pepsine, | - - - - | drs. ij. |
| Soda bi-carb, | - - - - | drs. iv. |
| Soda Phosphate, | - - - | drs, iv. |
| Theriaca, | - - - - | oz. iij. |
| Aq. Flora Aurant, | - - | oz. j. |
| Syr. Tolu, | - - - - | oz. iv. |
| Aq. Distil, | - - - - | ad oz. j.—M. |

*Perchloride of Iron in Paralysis of the Bladder.*—Dr. C. H. W. Parkinson, (Med. Times and Gazette) reports the case of a man suffering with paralysis of the bladder and its inconveniences, relieved and cured by injecting six ounces of a weak solution of tincture of iron in the bladder and allowing it to remain half a minute. The usual remedies had failed in this case before using injection.

LINIMENTS.—We subjoin, for the benefit of the readers of THE COMPANION, and especially rural practitioners, a couple of formulæ for liniments, which need only to be tried to insure success:

No. 1.

| | | |
|---|---|---|
| R.—Spts. Turpentine, | . . . | oz. viii. |
| Camphor to Saturation. | | |

Let stand 24 hours, and filter. The addition of camphor enhances the local use of the turpentine in its well known wide range of therapeutical application. As a resolvent for indurated glands and enlarged spleen, it will be found but little inferior to any of the preparations of iodine.

No. 2.

| | | |
|---|---|---|
| R.—Camphorized Turpentine, | . | oz. iiiss. |
| Rose Water, | . . . . | oz. ii. |
| Acetic Acid, | . . . . | oz. ss. |
| Vitellum Ovi, | . . . . | oz. i. |
| Oil of Lemon, | . . . | gtts. vi. |

First rub the lemon oil with the egg, gradually add the turpentine, and lastly, the rose water and the acid, in the order named. Bottle and let stand a few days, occasionally shaking well. The result is a very neat, cream like, liniment, which will serve as the basis for many other compounds as the judgment may dictate. It will be seen that this is an improvement on the celebrated St. John Long's Liniment, so highly alluded to by Dr. Graves, of Dublin. A few grains of acetate morphia dissolved in the acid, and chloroform added in the proportions of a drachm or two to an ounce of the liniment makes a very superior chloroform liniment. Common water may be used in lieu of rose water, and the lemon oil may be left out, but for pleasantness and efficiency, we would advise confirmity to the above formula. Again, 20 drops of croton oil rubbed with the egg in place of the lemon, gives an excellent examthemic liniment. But, enough to say, the ingredients of the mixture will readily suggest to the mind of every intelligent physician the great variety of uses to be derived from this liniment. Every country practitioner who is compelled to do his own compounding, should prepare it by the quart, as it improves by age.

JOHN R. HODGE.

*Holly Springs, Ark.*

RIGIDITY OF OS UTERI.—Dr. Theo. N. Rafferty, (Am. Practitioner) in an article upon Gelseminum Sempervirens, says: "In cases of protracted labor from rigidity of the os uteri, it is probably as near a specific as any other article of the materia medica.

*Chloride of Ammonium in Chronic Dysentery.*—Dr. Stewart (Brit. and For. Med. Chir. Review,) highly recommends the above remedy in chronic dysentery. He advises the continuance of the administration of the drug for some time after the disappearance of the symtoms.

*Dyspepsia.*—The following formula, suggested and much used by Dr. D. W. Hammond, of Macon, Ga., will be found worthy of trial, especially in sudden cases of indigestion accompanied with acidity. Dr. Hammond thinks no single remedy superior to this as a corrective in certain forms of dyspepsia. We have used it with great advantage:

R.—Pulv. Rhei,
Sup. Carb. Soda, - - aa. dr. ij.
Carb. Potass., - - - - scru. ij.
Aqua Camphor, - - - - oz. viij.—M.
S.—A tea-spoonful 15 minutes before eating.

Liquor Sedativus.—This is a combination often prescribed by English physicians in diseases complicated with febrile symptoms:

R.—Tinct. Opii Campor;
Spts. Æth. Nit. dulc.;
Spts. Minderri;
Syr. Simpi.;
Aq. Camphore, aa. part. æq.
M. et. ft. solutio. Dose: A tea-spoonful.

To increase the therapeutic effect of this mixture, 2 fl. oz. of tinct. gelsemini, or 1 fl. oz. of tinct. verat. vir. are often added to four ounces, to meet particular indications.—*Boston Journal of Chemistry.*

*Tenea Capitis.*—

R.—Carbolic Acid, Crys.,
Sulph. Sodæ, - - - aa. scru. j.
Sulphur Sublim., - - - - dr. j.
Adipis, - - - - - oz. i.—M.
S.—Apply twice a day after washing with soap and water.

Palpitation of the Heart.—Dr. Rafferty (Am. Practitioner) says, gelseminum is of unquestioned benefit in many cases of functional disease of the heart, characterized by the usual feelings of smothering and palpitation.

Creasote in Cholera.—Through the columns of the Medical Times I beg to ask the attention of physicians to the use of creasote as a remedy in cholera.

I have used it continuously for nearly twenty years in all stages of dysentery and diarrhœa, with great satisfaction to myself and benefit to my patients; relying almost entirely on its curative effects. I administer it to adults in doses of four drops every hour and a-half or two hours, combined with about twenty grains of bicarbonate of soda or potassa, mixed in syrup or honey. More recently I have substituted for the carbonates about eight grains of chlorate of potassa, in each dose, and in my practice have very rarely added an opiate or astringent to the mixture. I do not, however, consider the administration of opiates and astringents inappropriate in severe cases, nor do I advise the use of creasote in lieu of these medicines or other general treatment.

From my experience in the use of this medicine in these diseases, I am led to believe that it is not unlikely that in sufficiently large and often-repeated doses it will be of great service in the treatment of cholera, and perhaps that it might prove a valuable prophylactic, in doses of one or two drops to each glass of drinking water.

I have frequently admistered it in typhoid fever, with apparent advantage to my patients, and am satisfied that in the bowel affections incident to the life of armies in the field it would make the count stronger on the roster "for duty."

The object of this communication is to ask a fair test of this remedy by physicians who have to treat cholera.—*G. B. Latigue, M. D.*

For Nasal Catarrh.

| | | |
|---|---|---|
| R.—Quin. Sulph., | . . . . | grs. vi. |
| Pulv. Opii, | . . . . | grs. iv. |
| Ferri Per Sulph., | . . . . | grs. vi. |
| Lycopodii, | . . . . | dr. iij.—M. |

S.—To be used as a snuff in nasal catarrh. Should the discharge be offensive, add gtts. iii of carbolic acid.

Cystalgia.—In a case of a child one year old affected severely with Cystodynia, one drop of the Fluid Extract Gelseminum gave not only relief but a cure in 2 days.

Irritative Fever by Dentition.—Is often successfully treated with a-half ounce of fresh Chlorine water to 3 fluid ounces of Fennel water and give whereof a tea-spoonful every hour.

EPILEPTIC MANIA.—Dr. Frank A. Ramsey, (New York Medical Journal,) in a case of epileptic mania gave the following prescriptions with satisfactory results:

R.—Bromide Potass., . . . grs. xxx.
Chloral Hydrate, . . . grs. xv.—M.

S.—To be given when boisterous.

R.—Bromide Potass., . . . grs. xxx.
Fld. Ext. Cannabis.
Indica, . . . . gtts. xxx.—M.

S.—This quantity to be taken regularly, morning, noon and night. Under its influence the exhibitions of mania were very much modified.

CATARRHUS VESICÆ.—The following relieved an obstinate case in two weeks:

R.—Fluid Extract Buchu,
Fluid Extract Uva Ursi, . aa. oz. ss.
Fluid Extract Gelseminam, . dr. i—M.

S.—50 drops 5 times daily.

*Formulæ for Neuralgia.*—Dr. J. Hale (Am. Practitioner,) recommends the following:

R.—Quin. Sulph., - - - - gr. xxx.
Acid Arsenosi, - - - gr. j.
Ext. Belladonna, - - - gr. v.
Morph. Sulph., - - - gr. iij.—M
Ft. Pil. No. xv.

S.—One every four hours.

R.—Liq. Potas. Arsen., - - - dr. iv
Tr. Belladonnæ, - - - - oz. i
Tr. Aconiti rad., - - - - dr. iij.—M

S.—Twenty drops in water, three times a day, after meals. This, Dr. H. says, has proved very efficacious in some very obstinate cases in hands.

STICKS OF MITIGATED CHLORIDE OF ZINC.—Ordinary chloride of zinc is unsatisfactory as an escharotic, because of its hygroscopic tendency. M. Bruns states that this defect may be overcome by fusing two parts of the chloride of zinc with one of chloride of potassium, and casting in sticks,—the latter to be wrapped in tin foil.—*Practitioner.*

*Syphilis.*—Dr. F. T. Bumstead, in an interesting article in the American Practitioner, upon "Hints on the treatment of Syphilis," says: "I do not hesitate to give half a grain or a grain of the protiodide of mercury, combined with two grains of extract of gentian, in the form of pill, at noon after eating, and twenty, thirty, or fifty grains of the iodide of potassium morning and night; but I much prefer to administer the same quantity of the iodide three times a day, and to rub about a drachm of mercurial ointment into varying portions of the integument at night; at the same time directing the patients not to wash off whatever of the salve may remain, and to wear the same underclothes night and day. After the lapse of a week or ten days the patient is directed to cleanse the whole surface of the integument with hot water and soap, and to change his linen. The iodide is, however, to be continued, and the inunction to be repeated at intervals of a week or a fortnight, according to the exigencies of the case. The iodide of potassium should be given after meals, largely diluted with water (in not less than six or eight ounces of fluid, to which one or two drachms of the extract. sarzæ fl. comp. of the United States Dispensatory may be added.)

Speaking of the value of iodide potassium, he says: "The most heinous of all is an ignorance of the dose of the iodide of potassium requisite to give this agent its full effect and to test its power. By many men doses of two, five, or seven grains, given three times a day, are regarded as the utmost limit, beyond which it is unnecessary to go. If the symptoms do not yield to this treatment it is concluded that the iodide is not the remedy, and something else is tried! To think of a patient suffering with the nocturnal agony of periostitis, or threatened with the destruction of the palate or of the nose, being thus tampered with is almost enough to make one's blood boil. Why, the iodide may be used with safety and must be used, if its full effect is to be attained, with an unsparing hand. Relief will be had and important organs will be saved by giving one hundred grains a day, when the disease only laughs (metaphorically speaking) at fifteen or twenty! Patients find this out themselves when you have not stinted them in the use of the remedy; and will tell you, as one of my patients with syphilitic necrosis of the ulna recently did me, that forty grains three times a day had no effect, while fifty three times a day were at once followed by a manifest improvement. The iodide of potassium has been given with impunity in the quantity of two or three ounces in the twenty-four hours for several weeks and even months, but this amount is unnecessarily large. I have never had occasion to exceed three drachms a day; and from a drachm and a half to two drachms is usually sufficient."

*Hypodermic Injection of Quinine.*—This subject continues to excite attention, if we may judge from the communications that appear in our contemporary the Indian Medical Gazette. The civil surgeon of Ahmedabad states that he has administered quinine by way of hypodermic injection for upwards of two years, and over a hundred times; and that this experience enables him to speak of the advantages of this method, in point of economy and efficacy, with a certain degree of confidence. The dose he has generally employed has varied from two grains and a half to three grains, dissolved in four minims of dilute sulphuric acid and fifteen minims of water. In most cases the first dose was quite sufficient to put a stop to any further attack of fever; and in some it has benefitted the patient by prolonging the period of intermission. His experience of it in remittent fever is much more limited, but, on the whole, favorable. But, above all, the most markedly beneficial effects of the hypodermic injection of quinine may be observed in cases of brow-ague or hemicrania of malarious origin. In no instance does he remember its having failed, either in his own or his assistant's hands, to give rapid relief, which in some cases proved to be of a permanent character where only one injection had been employed.—*London Lancet.*

*Chronic Intestinal Catarrh.*—Dr. Kjelberg, of Copenhagen, recommends as a drink in this affection, in children who can not bear the customary dietetic treatment (such as milk, beef-tea, broth, raw flesh, Liebig's soup), "Liebigs's Vienna extract of malt." A desert-spoonful, dissolved in a cup of warm milk, is given three or four times a day. It is usually taken without objection, and is borne well. Experience has shown that the remedy has a good effect on the character of the dejections, as well as on the general condition of the child. While the treatment is more especially useful in children who have been weaned, Dr. K. believes it also beneficial in children at the breast who suffer from this trouble, and cites in support of this opinion the experience of Dr. Ahelin, of Stockholm.—*Schmidt's Fahrbucher.*

*Administration of Cod-Liver Oil.*—The following directions for the pleasant administration of cod-liver oil are given in the *Lancet* by Dr. Aubrey Wicks: "Direct the patient to place a few grains of chloride of sodium on the tongue before taking the prescribed dose of oil, (a piece of bread may be eaten after, if preferred.) With this simple adjunct patients who before rejected it will take the oil with apparent relish, describing the taste as that of herring or sardine."

Ascarides Removed by Hyposulphite of Soda.—Dr. Webb reported (Mich. University Medical Journal) a case of ascarides treated successfully with hyposulphite of soda. This case had been treated previously by aloetic cathartics and by various injections, but no permanent relief had been afforded, the trouble usually returning in about five days at longest. At first the hyposulphite of soda was used by injection, but afforded no more relief when given in this way than the remedies formerly used, he had ordered it taken by the stomach three times a day, the doses being sufficiently large to produce a slight catharsis. Relief was afforded at once, and the patient had had no recurrence of the trouble, though it had now been three months since the first exhibition of the remedy. The use of the salt was continued some time after the relief of the difficulty, to insure, if possible, a permanent effect.

*Starch as a Vehicle for Injections.*—Dr. George H. Bixby, at a meeting of the Gynæcological Society of Boston (Gyæcological Journal), presentad a new vehicle for vagina injections, namely, starch. Its use was suggested to him by seeing it so extensively employed by Prof. Hawley, the eminent French Dermatologist, in the form of baths, punctures, &c., in diseases of the skin. He thinks it is soothing, cleansing and healing. He uses it as follows: To one-half pint of thin boiled starch, add one-half teaspoonful of pulverized chlorate of potash, and three or four teaspoonfuls of glycerine. Use by injection every night before retiring, twice per day in urgent cases. He uses it also in nasal douches, injections of ear and bladder, and in chronic affections of the rectum. Sulp. zinc, plb. acetat, or other astringents may be used instead of chlo. potash.

*Tape Worm.*—Dr. Betz recommends the expulsion of tapeworms, even in the earliest age, for fear of the probable development of echinococci. He was successful in a case of a child six months of age. Parts of the worm had been voided five months before. He gave the half of the following mixture: Kamala-kousso, pulv. r. filic. maris *aa* half drachm, mell. despumat five drachms, aqua anisa three ounces; a teaspoonful every three hours.—*Memor., Ind. Jour. of Med.*

*Ether Spray to Spine in Anœmia.*—Dr. John Rose reports the case of an anæmic girl, aged thirteen years, who had chorea following rheumatism, who was successfully treated by ether spray, applied along the spine for four or five minutes each time; and after fifteen sittings, a very marked improvement took place, followed by complete recovery.—*Med. News.*

*Iodine.*—Dr. Schumidt details many interesting cures of passive congestions by iodine. Its indication, for instance, in nervous debility of females in beginning and ceasing puberty, during pregnancy and lactation, after long sickness, heavy loss of blood, weak nutrition, etc. But plethora, nervous rigor, irritation or inflammatory affections of the brain contra-indicate the use of iodine. The physician must be very careful in regard to the dose; one drop may be useful, but another one two hours after might excite great nervous trouble. This is the cause of the different opinions about it. Dr. S. also recommends iodine in vomiting and diarrhœa of pregnancy, and chronic diarrhœa connected with debility. Iodine was successful in old and apparently hopeless cases. The dose was one drop of the tincture or one-tenth of a grain in pill form every two hours. Dr. S. has used iodine for several years, in cholera morbus, in doses of one to two drops every ten to fifteen minutes. The worse the case the larger the dose. He saw the same excellent effect in cholera asiatica; the tincture was diluted with alcohol and an aromatic water, to be taken in teaspoonful doses.—*Berlin Klin Wochensch—Ind. Jour. of Med.*

*Dysmenorrhœa.*—In inflammation of the womb and in dysmenorrhœa, cold injections are not recommended by Despres. Violent bleeding, metritis, and pelvi peritonitis may result. The anæmia at first gives way to congestion, heat, and pain. Warm injections of about 90° to 100° work like warm poultices in inflammation; they soften and soothe the parts. Mr. D. observed the same effect in cases of dysmenorrhœa.—*Bulletin de Therap—Ind. Jour. of Med.*

*Treatment of Otorrhœa by Spirit of Wine.*—Dr. F. E. Weber (Berlin Klin. Woch., January 9, from Med. Times and Gaz., February, 1871, p. 239) states that spirit of wine is the best topical application in cases of otorrhœa uncomplicated with caries or polypus growth. The ear must first be well syringed out; then, the patient lying down, as much of the pure spirit as the ear will contain is poured in and allowed to remain five minutes. After the spirit flows out, the meatus must be dried and plugged so as to prevent the access of air. At first it is to be applied three times a day, then twice, and is to be continued for some time after the otorrhœa has ceased.

*Vomiting a Prominent Feature of Kidney Disease.*—Dr. Calvin Ellis, Boston, Mass., (*Boston Med. and Sur. Jour.*), reports three cases of disease of the kidney, in which vomiting was the sole symptom.

*Digitalis in Hemorrhage after Abortion.*—In the proceedings of the Gynæcological Society, of Boston, Dr. J. F. Gould, of Boston, reports the following case: "The patient was under the care of a midwife. The hemorrhage was excessive at times, so much so that combing the hair, or rather having her hair combed, she would faint while in the horizontal position, and had repeated attacks for eight weeks. The remains of the placenta were removed by me after the first attack, one week after the ovum had come away. I kept her feet elevated about fifteen inches higher than her head. These 'gushes' of blood were always controlled by gtts. x. tinct. digitalis, three times daily. Ergot was vomited in the fluid and solid form. The digitalis gave great relief to her weak heart."

*Bromide of Potassium in Croup.*—S. B. Kieffer, M.D., Carlisle, Pa., (*Chicago Med. Jour.*), cannot, after experiments from good authority, regard the bromide of potassium as *a solvent*, so to speak, of false membrane; but he does believe, and on this principle he has prescribed it, that just in proportion as it is a *sedative* to the cerebro spinal system directly, so it is a stimulant, indirectly, to the nerve-filaments and circulation of the throat; and, as the inflammation in membranous croup is usually, if not always, of the asthenic character, it has the power, by its specific action, of equalizing the circulation and arresting the fibro-albuminous deposit. And when the disease is not too severe, or has not progressed far, the system thus, by its own inherent power, will be equal to the task of repairing the evil. His combination is about as follows, for an infant:—R. Bromide of potassium, gr. xx.; chlorate of potassa, gr. x.; ipecac, gr. j.; ext. of liquorice, f. dr. ss; water, f. dr. ii-s M. S., a teaspoonful every hour.

Dr. W. W. Dale, (*Med. Times*), who has been using the same treatment, informs us that membranous croup, spasmodic croup, and laryngitis, alike have lost for him nearly all that dread and anxiety with which he once met them.—*Med. Record.*

*New Treatment for Small-Pox.*—Dr. J. J. Garth Wilkinson, of London, England, (*Canada Lancet*), has called the attention of the medical world to a new method of treating small pox, which he has tried in four cases of varied degrees of violence, with complete success. In these cases he used *hydrastis canadensis* and *veratrum viride*, both internally and locally as a lotion. The former, he says, extinguishes the varioloid poison, while the latter subdues the inflammation and primary fever. In regard to diet, he advises a judicious use of brandy and water, claret, Carlowitz or Hungarian wines (port, when the patient has begun to amend), beef-tea, and in consequence, fruit. He claims for this treatment that it abridges the duration of the disease, makes it almost painless, subdues the inflammation and primary fever, annuls the secondary fever, checks pustulation, prevents itching and stench, and saves the patient from any but the slightest pitting. He also claims for the *hydrastis* that it is an effective prophylactic or preventive to ward off the approach of the disease. The plant named *hydrastis canadensis* is found within the limits of New York State, and probably elsewhere in the United States and Canada. The plant is popularly called orange-root, and sometimes yellow puccoon, but it must not be confounded with another plant commonly called puccoon.

To quiet nervous restlessness, both during the course of the disease and the period of convalescence, he advocates the use of bromide of potassium.—*Med. Record.*

*Placenta Prœvia.*—Dr. E. R. Willard, of Wilmington, Ill., (*Chicago Med. Examiner*), publishes an interesting history of six cases of placenta prævia which came under his observation. He finds that the application of a sheet—the same as for *paracentesis abdominis*—answers admirably in making pressure upon the abdomen, for the purpose of restraining internal hæmorrhage. The external flow can also be more effectually controlled by continuous firm pressure with a proper fitting tampon. In the absence of anything better, he has found a small sized glass speculum, properly padded, and inserted wrong end up, to answer a good purpose. This procedure necessitates the constant personal attention of the physician until the accouchment is complete.—*Med. Record.*

Chorea Treated with Strychnine—In a severe case of chorea, reported by Dr. Hayden (*Brit. Med. Jour.*, May 20,1871), the patient, a girl 1[illegible] years of age, took the one-twentieth of a grain of strychnine in mixture three times a day. This treatment was continued until slight rigidity of the muscles at the back of the neck was observable, with the effect of moderating the contortions of the shoulders and trunk. She was afterwards put on half-drachm doses of the syrup of the triple phosphate (strychnia, quinia and iron) three times a day. This produced the most beneficial results. A recovery was effeted in about six weeks—not a long time, when the severity of the case is taken into consideration.—*Medical Times.*

Bromide of Lithium.—Dr. W. A. Hammond, advises the use of the Lithium Salt in preference to the Bromide Potassa, in all cases where speedy effect is desired or demanded.

Veratrum Viridi an Antidote to Opium.—Dr. E. H. Sholl, of Alabama, communicates a case of poisoning by morphia, which was cured by veratrum. The patient, a negro boy, aged 15 years, had typhoid fever, and took an overdose of morphia, which had been prescribed for hiccough. It was followed by stertorous breathing, contracted pupils, and so forth. His mouth was pried open, and gtt. xviij. Norwood's tincture poured in with two ounces of brandy. In one hour, every symptom of poison had vanished.—*Medical and Surgical Reporter.*

*Medical Use of Carbolic Acid.*—Dr. N. S. Davis, Chicago, Ill., (*Chicago Med. Examiner*), in his "Report on the Medical Use of Carbolic Acid" to the Illinois State Medical Society, refers to two cases of cancerous disease and ulceration of the os and neck of the uterus which were much relieved by using the solution of carbolic acid internally, at each meal-time, and a stronger solution twice a a day as a vaginal wash. They were kept comparatively comfortable for many months, but the effects were only palliative. A neighboring physician informed him that he had a case of cancerous disease, of well-marked character, that had been kept stationary and the patient comfortable more than twelve months, under the constant use of carbolic acid. Dr. Davis has also used it with temporary benefit in two cases of cancerous disease of the stomach. He does not regard carbolic acid as a specific for the cure of any form of disease, but from its mildly sedative influence on the organic nervous system and mucous surfaces, coupled with strong antiseptic properties, it is admirably adapted to meet certain indications that arise during the progress of a great variety of disease.—*Ibid.*

*Carbolic Acid in Diphtheritic Affections.*—Dr. F. C. Hotz, Chicago, Ill., (*Chicago Med. Journal*), wishes to draw the attention of the profession anew to this remedy, by reporting a number of cases of diphtheria which, during the last winter, he treated with the most gratifying success with carbolic acid, He used for this topical medication the acid in the following form: R. Acid. carbolic cryst. alcohol, aa dr. j.; aquæ, dr. v.; tinct. iodin., dr. ss. M. This makes a perfectly clear, transparent mixture, of a brown-red color, which soon, however, passes over into a pale-yellow. The combination with iodine and alcohol effectually moderates the unpleasant smell of the carbolic acid, and, increases its antiseptic effect. This solution was applied to the diphtheritic exudation three or four times in twenty-four hours, by means of a camel's-hair brush. In adults and older children, it was in a diluted form (15 to 20 drops to a cup of water) used also as a frequent gargle, and for injections into the nostrils if the nose was implicated.

*Digitalis as a Remedy*—Dr. N. D. Cone, (Medical and Surgical Reporter) thinks that digitalis, as a remedy in certain forms of heart diseases, had never been, nor is now fully, appreciated. This, he thinks, is due to its having been given in improper cases, and, again, fearfully in consequence of its so-called accumulative action. The Dr. has given it successfully in very large doses. He ususually commences its use with 10 to 15 drops every 4 hours, increasing gradually until the proper dose is reached, which is frequently not attained short of half a drachm to drachm-doses, 3 times a day. He has treated cases of "heart disease" in this way, with uniform success.

*After-Pains.*—Dr. Wellington, (Boston Medical and Surgical Journal,) says, that he gives a tea-spoonful of the fluid extract of ergot, in water, directly after labor, in all cases. It diminishes the intensity of the after-pains, except those immediately following the termination of labor.

*Vomiting of Pregnancy Cured by Change of Position.*—Dr. Read, (Boston Medical and Surgical Journal) reports a case in which, after using everything to no purpose, he arrested the nausea and vomiting by placing the patient in bed and elevating the hips as high as possible above the level of the shoulders.

*Gastralgia Treated by Electricity.*—Dr. Mierques (Lyon Medical) applies in gastralgia a plate of zinc on the epigastrium, under which there is placed a piece of linen moistened with vinegar and water, and a similar disc of copper between the shoulders. These are united by a conducting wire, and kept on by a bandage. These are useful, he says, in neuralgia, and cramps of the lower extremities, etc.—*The Doctor.*

*Chorea.*—We find in the *Astliches Literaturblatt* for July 7th, M. Wenz treated a girl of seventeen years of age, who had not hitherto menstruated, but who had been for several weeks affected with chorea of the right side, by local anæsthetisation of the skin over the spinal column. It suspended consciousness, giving rise to a kind of catalepsy. It was repeated for several days, with the result that the chorea was cured. M. Wenz suggests a trial of this plan in tetanus.—*The Doctor.*

Strychnia in Chorea.—Dr. W. A. Hammond states that he has found strychnia an unfailing remedy in chorea. In connection with the strychnia he also usses ether spray along the spine, a cure resulting in every case in two weeks.

*Bromine and Kerosene Applications in Cancer.*—At the recent meeting of the British Medical Association, (Lancet,) Dr. Wynn Williams read a paper "On the Treatment of Cancer of the Neck of the Uterus and Allied Structures by the Injection and Application of Bromine." "The author commenced by making some remarks on the spontaneous removal of malignant tumors, from the study of which he was led on to the injection of bromine into cancerous tumors of the uterus and other parts. He stated that the eight cases published in the last volume of the Obstetrical Transactions still continued well. He entered into full details as to the manner of injecting these deposits, and the care required in the use of bromine both as an injection and application, and that before its use the surrounding parts should be well protected by a solution of soda. He exhibited the various instruments he had had made for the injection of bromine. He gave the history and successful treatment of a well-selected case of medullary carcinoma of the uterus in the state of disintegration and ulceration by this method. He also gave the particulars of a case of epithelioma of the lower lip which had been previously removed by operation. On the return of the disease the patient was sent to Dr. Wynn Williams, who, by two injections of bromine, caused the entire and, so far, permanent removal of the disease."

The editor of the Medical Press and Circular, says, Dr. Sedgwick S. Cowper writes, that he has found "the vegeto-mineral oil, known in Australia as kerosene, wonderfully curative in the treatment of ulcerated and cancerous wounds."—*Medical Cosmos.*

*Cholera*—A writer in El Pabellon Medico maintains that opium is as successful in cholera as quinine is in ague, and that it should be given in doses proportioned to the gravity of the case. He therefore has recourse to full doses of opium frequently repeated.

The following formula for cholera pills is that of M. Bourgone:

| | | |
|---|---|---|
| R.—Tannate of quinia, | - - | gramme, i |
| Powdered opium, | - - | centigrs. v |
| Essence of aniseed, | - - - | gtts. ii |

Simple syrup. to make 10 pills; which may be taken in the course of one or two hours.—*The Doctor.*

*Iodide of Calcium.*—Dr. Goodman (Canada Lancet), at a recent meeting of the Medical Society for Mutual Improvement, spoke in favor of the use of iodide of calcium as a remarkably mild and efficient alterative; it appeared to him to be more easily assimilated in disordered states of the stomach than any other iodide; he had used it lately with marked effect in diseases of the stomach and bowels in the strumous diathesis.

*Pressure on Abdomen in Uterine Hæmorrhage.*—Dr. J. H. H. Burge (Richmond and Louisville Medical Journal,) "contributes the history of a case of placenta prævia attended with profuse hæmorrhage. He found a small margin of the placenta presenting. The liquor amnii had completely drained away, so that the outline of the child could be made out through the abdominal walls. The examination excited some pain, and caused considerable hæmorrhage. Observing that during the pain the pressure of the child's head upon the placenta controlled the flow, he, in the intermission, grasped the uterine tumor and pressed steadily in the direction of the os. This was continued with perfect success for two and a half hours, when a living child was born, and the placenta followed without loss of time. During all this time, if the pressure was relaxed while the uterus was inactive, hæmorrhage was sure to follow."

In connection with external pressure in placenta prævia, Dr. J. H. Traylor, of Cape Girardeau, Mo., (Medical and Surgical Reporter) recommends the rupture of the membranes and the free use of ergot to produce tonic contraction of the womb and bring the presenting part of the fœtus in direct contact with the bleeding surface.

*Chloro-Acetic Acid in Fibrous Growths, Etc.*—Dr. W. H. Atkinson says (Dental Cosmos,) this agent "has a strong affinity for dead connective tissue, epithelial scales, indurations, warts, and fibrous growhts. For correcting unhealthy and brawny faces, for which 'enamels,' 'bloom of youth,' etc., have been resorted to such an execrable extent, and producing a beautiful skin, these acids, judiciously used, stand unrivaled, dissolving off the old scale, and favoring the growth of a new supplay."—*Med. Cosmos.*

Ol. Terebinth. in Chronic Ulcerations.—J. Fayrer, M.D., C.S.I. (Medical Times and Gazette), alludes to the benefit that may be derived from the internal administration of small doses—20 to 30 drops—of ol. terebinth., given at intervals of four or six hours for some days, in the treatment of chronic ulcerations of an indolent character. He first saw it used by the late Dr. Gilbert King, Inspector-General of Hospitals, R. N., at Bermuda, so long ago as 1844. The effect on the capillary circulation is most marked, and he has frequently seen it succeed in promoting healthy granulation in most obstinate cases of chronic ulceration. To prevent strangury, it may be advantageously combined with nitric ether, and occasionally with tincture opii.

Ergot in Spinal Congestion.—Dr. W. A. Hammond recommends ergot in spinal congestion, having found it of decided benefit.

*Wet Sheets in Diarrhœa.*—Oppenheimer employed this treatment in twenty cases of rapid diarrhea in children, from fourteen days to four years old, with three deaths and seventeen recoveries. In two cases of chronic diarrhea there was no result. He used sheets wet in water at 50° to 55° Fahr., in which the little sufferer was enveloped and then covered with a blanket, which was kept on until perspiration set in—say from half an hour to an hour. Internally the patients were given ice-water, broth, barley-water, milk, wine or brandy, but no medicine. As soon as perspiration occurred the packing was removed, the child wiped dry, and cloths dipped in cold water laid upon its abdomen, and renewed as fast as they grew warm. Where the diarrhœa persisted the packing was repeated within an hour. If cerebral congestion occurred, the packing was discontinued and ice applied to the head. The treatment seems suited only to acute attacks of diarrhœa in children.—*Schmidt's Fahrbucher.*

A Case of Nephritic Calculus.—Dr. Willien treated a case of nephritic calculus, (*American Practitioner*) attended with extreme suffering, by applying a blister over the loins, and giving internally a teaspoonful every three hours of the following:

| | | |
|---|---|---|
| R.—Act. potass., . . . | aa. . . | oz. ss. |
| Vin. colchic., . . . | | |
| Tinc. opii camph., . | | |
| Aq. cinnam., . . . | | |
| Spts. nitr. dulc., . . . . . . . | | oz. j. |
| Extr. fluid belladonna, . . . . | | dr. j.—M. |

The next morning the patient voided a phosphatic calculus weighing five grains.

*Reflex Epilepsy from Disease of the Ear.*—Dr. Schwarts communicates to the Centralblatt, May 20, 1871, the case of a young man in whom epilepsy seemed to be caused by disease of the middle ear. The countenance was expressive of suffering, there was slight paralysis of the left facial nerve, and the skin covering the mastoid process of the left side was red and swollen, and, when pressure was made upon it, it was found to be very sensitive. Dr. S. thought it right under these circumstances to perforate the mastoid process, in the hope that with the evacuation of the thickened pus that was retained in the middle ear the epileptic paroxysms would cease. After the operation there was no more severe seizures, and the patient's general health was very much improved, there having been no attack, at the time of reporting the case, for several months.

POLYGONUM HYDROPIPER.—J. S. Unzicker, M.D., Cincinnati, Ohio (The Clinic), recommends the saturated tincture of Polygonum Hydropiper (water pepper) as an emmenagogue. It was first recommended by the late Prof. John Eberle, in cases of amenorrhœa, in teaspoonful doses, three or four times daily. It was extensively used for many years by all the leading physicians of Cincinnati, and highly spoken of. This reputation it retained so long as the tincture was made of the right material, and the remedy given in the proper cases. But when it fell into empirical, both medical and pharmaceutical, hands, and was given indiscrimately in all cases, and often made of the wrong material, it had, as a matter of course, to fail in many, and in consequence, like many other good remedies, was laid aside and partly forgotten. Dr. Unzicker believes it is the safest and best remedy in cases where the general health is not impaired, but when the menses are checked by cold, mental emotion, or other like cases, where you wish to bring them on without fear of producing congestion to the head or chest, a sequence to be dreaded.

AN EFFICIENT DIAPHORETIC.—Dr. T. Osborn recommends the following diaphoretic as having been found useful in the region of Alabama in which he has practiced physic:

| | | |
|---|---|---|
| R.—Chloroformi, . . . . | } aa. . . . | oz. ss. |
| Spts. etheris nitrosi, . . | | |
| Tinc. opii camphoratæ, . | | |
| Vini antimonii, . . . | | |
| Aquæ, . . . . . . . . . . . . . . | | oz. ij. |

M. S. For adults, one table-spoonful every hour until the fever abates.—*American Practitioner.*

*Treatment of Pruritus Vulvæ due to Vaginitis.*—Dr. Lorrile Athill, in a clinical lecture (Dublin Medical Press, June 21, 1871), primarily states that puritus rarely occurs except as a symptom of inflammation of the mucous membrane of the uterus or vagina, and can only be relieved by curing these affections. If vaginitis alone exist, with a view of attaining this object and checking the pruritus which it causes, use in the first instance soothing applications, then stringent ones. Of the former, none can compare with infusion of tobacco. It should be made by infusing two drachms of the unmanufactured leaf in a pint of boiling water. He has never seen the least unpleasant effect follow its use, while the relief afforded is most marked.

Another mode of treatment of the greatest value, according to this gentleman, is glycerine. A roll of cotton-wool, with a strong thread attached to facilitate removal, and saturated with glycerine, should be passed into the vagina through a speculum, and allowed to remain there for twenty-four hours; this produces a copious watery discharge, which is often followed by very satisfactory results.

Syringing the vagina with a solution of borax in tepid water or infusion of tobacco is in such cases of great use. It should be used in the strength of three drachms to the pint, and injected by means of one of the continuous siphon syringes. To allay the intolerable itching, Dr. Athill has also been in the habit of recommending the patient, after she has sponged herself with warm water, to lay inside the labia a piece of lint soaked in a lotion composed of carbolic acid, ten grains, acetate of morphia, eight grains, dilute hydrocyanic acid, two drachms, glycerine, four drachms, and water, two to four ounces.

*Eryspelas.*—The following used as an external application by being brushed over the diseased parts, as often as necessary, has been found to exert a most decided and healthy influence:

| | | |
|---|---|---|
| R.—Tr. ferri chlor., | . . . | f. dr. ss. |
| Quin. sulph., | . . . | dr. ss. |
| Tr. cinchona, | . . . | f. dr. ij.—M. |

Use as denoted above.

In traumatic erysipelas it may be also used internally in proper doses, so as to give not less than 30 or 40 drops of Tr. ferri, every hour or two.

*Chlorate of Potash in Uterine Epithelioma.*—Dr. Ercoli (Doctor) says the chlorate of potash has been used successfully in carcinomo of the face. In a case of uterine epithelioma, after the diseased parts had been removed by the galvano-caustic, the neoplasom having attacked the parts above the cervix, injections of a saturated solution of chlorate of potash was used so successfully, that the lancinating pains ceased, as well as the serous cancorrhœa and the irritation.

*Acute Rheumatism treated by application of Nitrate of Silver.*—Dr. W. Furgus (London Lancet) gives a case of articular rheumatism in which he applied the stick-caustic to the whole surface of the joints with such success, that in less than two hours the pain had lessened, and in twelve hours had subsided. The pain never reappeared when the cuticle was removed.

*Succus Conii in Hydrocephalus.*—Drs. Echeverria and McDonald (Doctor) state that in a case of hydrocephalus every remedy failed to arrest the convulsions, except the succus conii.

*Prevention of Scarlatina.*—Dr. W. M. Searcy, in an interesting article (Nashville Medical Journal), thinks, if, when physicians are called to cases of scarlet fever from day to day, they will carefully examine the tonsils of those of the family who have not yettaken the disease, they will generally find the tonsils exhibiting more or less redness and enlargement. In all such cases he at once applies a solution of nitrate of silver—dr. i. to oz. i. distilled water—every day until signs of amendment are visible, and then every other day until the local disease disappears. When the glands are much enlarged and red, he gives a few grains of calomel, followed in a few hours by castor-oil, or a saline. He gives thirty-three instances of individuals who never experienced an attack, although directly exposed, after the employment of the caustic as above recommended.

*Cod-Liver Oil and Iodide of Iron.*—Dr. J. Cummiskey (Philadelphia Medical Times) says the following formula will be found very valuable, where the combined action of these medicines are sought:

| | | |
|---|---|---|
| R.—Iodide of iron, | . . . . | gr. lxiv. |
| Ether, sulphuric, | . . . | q. s. |
| Cod-liver oil, (clarified) | . . | o. j. |

Dissolve in a mortar, the iodide of iron in a slight excess of ether, and add the oil gradually, stirring with the pestle rapidly until the mixture is complete. Keep in a tightly corked bottle. It is a stable compound. The proportion above is one grain of iodide iron to one drachm of oil, which is the dose. It may be increased, however, to f. oz. ss. if necessary.

Rapid Vessication from Emplas. Catharid.—Dr. I. L. Van Zandt, communicates to the Richmond and Louisville Medical Journal a plan by which vessication is accomplished in three hours. He spreads the cerate on cloth; on the surface of the cerate a piece of soft paper, and saturates this with acetic acid, smooths it down on the plaster, letting it remain fifteen or twenty minutes. He then removes the paper and applys to part where vessication is desired.

Ergot of Rye in Insanity.—Dr. J. C. Crichton Browne (Practitioner) has been testing, for six years, the therapeutic value of ergot in various forms of insanity. He believes it to be of great value in three varieties: 1st. Recurrent Mania; 2d. Chronic Mania, with lucid intervals, and, 3d. Epileptic Mania. In all these forms it lengthened the internals of excitement, and occasionally prevented their occurrence entirely.

*Chronic Dysentery.*—Dr. J. S. Warren, Louisville, Ky., (Richmond and Louisville Medical Journal) says: In chronic dysentery we have good effects from the following prescription:

R.—Vin. ipecac,
Tr. nux. vom., . . . aa. dr. ij.—M.

S.—Twenty drops three times daily, either before or after meals, as it best agrees with the patient. One patient, he says, who had taken everything that is usually given in dysentery, was completely cured by the above. The effect of the remedy is to produce temporary catharsis—but which is easily controlled.

*Hyposulphite of Soda in Variola.*—W. A. Corwin, M. D., (Medical Record,) has used this drug in twelve cases of small-pox. It was employed by him *only* in the premonitory fever, giving a supporting treatment alone, *after* the eruption appeared. He speaks of its remedial power, in this disease, very highly.

*Phthsis Pulmonalis.*—Dr. A. Nasse, (Jour. Mat. Med.) says he has found the following combination to act very happily:

R.—Cod-Liver oil, . . . . oz. vi.
Syr. squill, . . . . oz. i.
Bromo-chloralum, . . . dr. j.—M.

S.—Tablespoonful three times a day.

HÆMORRHOIDS.—Sir Henry Thompson still favors the ligature in internal pile. Mr. Heath secures it with Smith's clamp, cuts and applies the cautery.

*Treatment of the Suppurative Stage of Burns.*—The following is recommended:

R.—Bees-wax, melted and strained, . . f. oz. i.
Flax-seed (or sweet) oil, . . f. oz. ij.
Tannic acid, . . . . . dr. j.

Mix as follows: Put in a clean tin vessel and melt the wax; add the oil and stir until they are thoroughly incorporated with each other; then set off the fire and continue to stir at short intervals until cold; add the tannin immediately after setting off; add six or eight drops of fluid carbolic acid to the ounce.

S.—Spread on lint or soft cloth and apply to all the denuded and burned parts, securing, if necessary, by bandage.

---

THE GEORGIA MEDICAL COMPANION should be taken by every physician in the land.

Remedy for Removing Biliary Calculi.—Dr. Babbitt, (Michigan University Medical Journal) a sufferer from biliary calculi, used the following remedy, which he calls "specific":

| | | |
|---|---|---|
| R.—Tr. Opii, | . . . . . . | f. dr. iij |
| Tart. Anti., | . . . . - | gr. ij |
| Aq. Tepidæ, | . . . . . | oz. iv.—M |

S.—Inject per anum as soon as the attack is fairly made, and, three or four hours after, swallow from a teacupful to half a pint of pure olive oil. He remarked, that soon after he had swallowed the oil he passed over one hundred biliary calculi, fifty of these being as large as peas.

Ulcus Serpigenosum Sylpiliticum.—Dr. J. C. Ray (Am. Jour. Syph. and Derm.) reports a case treated, successfully and rapidly, by the following:

| | | |
|---|---|---|
| R.—Hydrag. Chlo. Cor., | . . . . | gr. ii |
| Potass. Iodide, | . . . . | dr. iv |
| Aquæ, | . . . . . | oz. iv.—M |

S.—One tea-spoonful, in a wineglass of water, after each meal.

R.—Ungt. Hydrarg. Ox rub.
Ungt. Simplic. equal parts.
M.
S.—To be applied to the ulcers.

Sulpho-bionate of Sodium in Dyspepsia.—Dr. Warren, (Gynæcological Journal,) states, that he has found great advantage from the administration of the sulpho-bionate of sodium in dyspepsia, especially that accompanying pregnancy. He administered it in doses of eight grains, three times a day, and noticed that it relaxed the bowels.

For Intermittent Fever.—Dr. J. Hale, (Am. Practitioner,) recommends the following:

| | | |
|---|---|---|
| R.—Quin. Sulph.. | . . . . . | gr. xx |
| Pil. Hydrarg., | . . . . . | gr. x |
| Piperin, | . . . . . . | gr. x |
| Pulv. Opii, | . . . . . . | gr. ij.—M. |

Fl. Pil. No. x.

S.—Two every three hours, beginning twelve hours before the expected paroxysm, to be followed by an aperient.

### PART III.

### Editorials, Correspondence and Miscellaneous.

### OUR THANKS,

Are due Drs. Jno. R. Hodge, of Arkansas, and C. B. Galloway, of Mississippi, for contributions to our Second Part. We trust they will continue on "this line," and that others will follow their example. Many a gem may see the light and prove a blessing to both, physicians and people, by our friends in this way. Friends, overhaul your case and prescription books, and give our readers the benefit of their contents. Will you? We also thank Dr. L. B. Bouchelle, for his favor which appears in the editorial department—as well as all other friends, whose names are withheld, for their letters, extracts from some of which wepublish in this number. Will not other writers "come up to the help" of truth, principle and law, "against the mighty?"

### DR. ROBERT BATTEY,

Gives us another of his interesting, instructive and highly practical articles in this number. We are sure the reader will be assisted by early adopting, in every case, the very practical and happy method of treatment employed by the Doctor, in intestinal obstructions.

### OUR SECOND PART.

We tender our thanks to those of our friends who have sent us contributions for this department of THE COMPANION. We desire to make the Second Volume very practical and valuable, in this respect. Will not our friends, everywhere, send in their "*little gems,*" as well as larger articles, for publication?

Have you a "pet" formula, or a favorite remedy for any particular disease? Send them in. Share your good "little things" with your brethren.

DR. W. T. TAYLOR.

The valuable article, on the hypophosphites in Cholera Infantum, published in our October number, was from the pen of the above gentleman, and not "T. Taylor," as the printer made it appear. We hope to here from the Doctor again very soon.

CHICAGO MEDICAL EXAMINER.

The office of the above-named journal was destroyed by the great Chicago fire. We are pleased to see that, phœnix like, it has risen from its ashes and that the October number—so long delayed—has been published. We sympathize with our brethren in this great calamity, and trust the generous hearts of the profession, everywhere, will be moved to action in their behalf.

QUININE AS AN OXYTOCIC.

Dr. W. A. B. Norcom, of N. C., in a private letter, asks: "Have you any experience with quinine as an oxytocic? In a letter just received from my friend, Prof. Thomas, of N. Y., he says he knows of no such power it possesses. My experience corroborates his views"

We have never seen any such power in quinine as an oxytocic—nevertheless many distinguished names are arrayed in opposition to the views expressed above. A question like this can alone be determined by carefully selected facts. We call the attention of our correspondent to an able and lengthy discussion of the subject, in the November number of THE COMPANION, by our medical friends of the Macon (Ga.) Medical Association.

QUININE IN CONVULSIONS OF CHILDREN.

We lay before our readers an article upon the treatment of the convulsions of children with large doses of quinine, from the pen of our Associate, Dr. E. H. W. Hunter, which appeared originally in the Savannah Medical Journal. The article has been revised by Dr. H., and therefore of greater value. We are promised another article upon the same subject, by our co-laborer. We see, by one of those coincidences which no one can explain, that our friend, Dr. Maxwell, of Mississippi, reports to the Lowndes County (Miss.) Medical Association a case of convulsions, in which he employed large doses of quinine, with gratifying results. Other gentlemen, of that Association also, regarded the quinine treatment of convulsions with favor. The respective fields of the professional labor of our friends are, doubtless, decidedly malarial, hence we should judge that the beneficial results of the quinine treatment were due largely, to that fact. Years ago Dr. Fenner, of N. O., gave large doses of quinine in all forms of convulsions—a practice enthusiastically followed by Dr. Cartwright.

ARTESIAN WELLS.

We will publish in our January number, an able and exceedingly interesting communication upon Artesian Wells, from the pen of one of the most scientfic and distinguished medical gentleman of the country—Dr. Lipscomb, of Columbus, Miss. It will be read with gratification and pleasure everywhere.

### OUR INDEX.

We close our first volume with a carefully prepared index. In looking over this, our subscribers will find an immense amount of valuable medical facts of the greatest practical importance to the active, busy practitioner. We have adhered strictly to the idea of condensing the medical literature of every part of the world, coming to our hands. To the extent of our ability we have faithfully winnowed the very best medical wheat from all sources. We have not succeeded to our satisfaction, in every particular, in carrying out our plan, but we have opened up the road, and feel better prepared to do so in the future. Of this, however, our subscribers must judge. The next volume, we hope to make greatly the superior of the present in every respect. Our corps of gifted and talented Associate and Corresponding Editors, with other contributors, will make the original department the equal of any journal in the country, and we, on our part, promise to collect the cream from the best medical literature of both hemispheres, for the use of our readers.

We are gratified to know that our efforts have been appreciated. Although the editions of The Companion have been large, yet several of the numbers have been exhausted, and it will be impossible now, to furnish a complete set to new subscribers. This we regret, however much we might congratulate ourselves upon the fact as an evidence of the appreciation of The Companion by the medical profession. This inability on our part, to supply back-numbers, will be regretted by many, and we would therefore, take occasion to call attention to the great importance of commencing the next vol. with the very first number, so that our subscribers may have a complete set ready for the binder at the close of the next year, and properly indexed for easy reference. Our publisher authorizes us to say that those of our subscribers who will obtain a club of five, will be entitled to their copies gratis, or in lieu thereof, will have their volume bound free of charge. We again ask our non-subscribing friends to look at the index of this volume. Please examine it carefully. We beg to ask them would they take *two dollars* for the imformation it would surely impart, of the very greatest importance to them as practitioners ? Thus for $2, and $1 50 additional for binding, we will be enabled to furnish our subscribers the best and cheapest bound medical periodical, we believe, in the country. One they would not relinquish for ten times that value. Go to work friends ! We want to see every physician in the land a subscriber. The poorest and humblest can easily pay the subscription price. A little trouble, a little solicitation on the part of our friends will make the subscription list of The Companion the largest in the Union.

At the suggestion of friends, we send to some of the best men of the profession, the present number as a specimen, with conditions they will understand upon its reception.

### A UNIFORM FEE-BILL.

Would it not be wise for the Georgia Medical Association to take the initiative in the matter of a uniform fee-bill for the State. It seems desirable that there should be one, and the Association seems to be the place for its birth and maturity. It is respectfully suggested to the members of the Association, to consider this matter.

It seems almost a necessity, to both the profession and our patients. Such a bill would manifestly curtail litigation and save money to both parties. Of course, there would be a different rate for city and country practitioners.

L. B. Bouchelle, M. D.

We invite the attention of the profession of the State to the above. The suggestions of our Associate should merit the earnest consideration of every medical gentlemen. We will say something on the subjects suggested, in our next.

