# Correction

**Published:** 1872-02

**Authors:** 


### CORRECTION.

The article on Liniments, in our December number, was from the pen of Dr. John Knox Hodge, of Holly Springs, Ark., and not Dr. J. R. Hodge, as the printer made it.

We thank Dr. Hodge for his little gems. They are the very things many of our readers wish to see—something not *wild* and *frothy*, but of practical value, as therapeutical remedies. They are worth more than pages of gaseous nonsense, in vain efforts to extort " general principles" therefrom. We trust others will follow the Dr. in this good work. Why not?

